# Falciform fat:femur length ratio provides a novel method for objective postmortem estimation of total body fat in overweight and obese cats

**DOI:** 10.1177/10406387211071078

**Published:** 2022-01-10

**Authors:** Cecilia Ley, Alexandra T. Leijon, Tora E. Nyberg, Lisa M. Lindström, Charles J. Ley

**Affiliations:** Departments of Biomedical Sciences and Veterinary Public Health, Swedish University of Agricultural Sciences, Uppsala, Sweden; Departments of Biomedical Sciences and Veterinary Public Health, Swedish University of Agricultural Sciences, Uppsala, Sweden; Departments of Biomedical Sciences and Veterinary Public Health, Swedish University of Agricultural Sciences, Uppsala, Sweden; Departments of Biomedical Sciences and Veterinary Public Health, Swedish University of Agricultural Sciences, Uppsala, Sweden; Clinical Sciences, Swedish University of Agricultural Sciences, Uppsala, Sweden

**Keywords:** adipose tissue, cats, multidetector computed tomography, obesity, pathology

## Abstract

Determination of the nutritional condition, including estimation of amounts of total body fat (tBF), at routine postmortem examination of cats is typically based on subjective visual assessment. Subjective assessment may result in uncertainties regarding degree of overweight, and objective methods that provide a numerical value reflecting the tBF could be valuable to accurately judge excess body fat. We investigated if the falciform fat pad weight (FFPW) was correlated to tBF and could be used to detect overweight and obesity in cats. The FFPW and the femur length (FL) were recorded at postmortem examination in 54 cats and the FFPW:FL ratio (FFR) calculated. Each cat was additionally assigned to a fat category (FC) according to subjective assessment. Computed tomography was used to determine tBF as the body fat percentage (%BF), the body fat volume (BFV), and BFV normalized to animal size (nBFV) in 39 cats. There was strong correlation between the FFPW and the BFV (*r* = 0.888) and between the FFR and the nBFV (*r* = 0.897). The correlation between the nBFV and %BF was very strong (*r* = 0.974). Using a lower FFR cutoff value of 3.5 for obesity and 1.6 for overweight, there was a discrepancy in FC between using the FFR and subjective assessment in 6 of 54 cats (11%). We conclude that the FFPW increases proportionally with tBF and that the FFR provides a method for objective tBF estimation. We suggest introducing the FFR to feline postmortem examination protocols as an objective estimate of tBF.

Similar to the situation in human medicine, overweight and obesity is a major health concern in small companion animals.^7,10^ By definition, *overweight* refers to a body composition in which the amount of body fat exceeds the optimal amount of fat for maintaining good health, whereas in *obesity* the overweight is of such magnitude that is likely to have serious consequences for the health of the individual.^22^ In cats, overweight and obesity conditions may predispose to musculoskeletal, respiratory, gastrointestinal, urinary, skin, and ophthalmic disease.^16,24,26^ Further, obesity exacerbates the predisposition to glucose intolerance in cats,^1,17^ and excess body weight is a risk factor for feline diabetes mellitus.^18,21,26^

Clinically, determination of the body condition in cats is often done using subjective, semi-quantitative graded point scales, based upon visual and palpatory evaluation that reflect subcutaneous and abdominal fat.^9^ Concerns have been raised that such methods could be subjected to non-standardized evaluation.^6^ Imaging, using dual-energy x-ray absorptiometry (DEXA) and computed tomography (CT), can be used to assess objectively the amounts of total body fat (tBF) in cats, but these methods are typically only used in research settings.^3–5,14,15,19^

Assessment of the nutritional condition is part of routine postmortem examination of animals, including cats.^12^ The graded point systems developed for clinical settings become less reliable postmortem because lack of muscular tone may affect both visual assessment of body contour and influence palpatory findings of subcutaneous and abdominal fat. Postmortem examination allows a more detailed assessment of tBF compared to clinical methods, given that multiple internal fat deposits can be assessed visually. However, because the postmortem estimation of amounts of body fat also typically is based on visual evaluation, determination of whether the animal is normal weight, overweight, or obese will still be influenced by subjectivity, and inter-observer differences are likely to occur.

Increased awareness of obesity-related disease in humans and animals and the One Health perspective of obesity^6,22^ highlight the need for methods that estimate tBF accurately. Standardized objective methods of assessing tBF could contribute to reduced inter-observer variability in the detection of overweight and obesity and facilitate communication among anatomic pathologists, clinicians, and students in regard to the nutritional condition of the animal. Further, objective estimation of excess tBF could provide strong support for the validity of subjectively assessed tBF in cases in which the animal welfare aspects of obesity are of interest.

We aimed to develop a method for objective detection of overweight and obesity at postmortem examination of cats. We investigated whether the amount of fat in the falciform ligament (i.e., the falciform fat pad) reflected tBF as determined by whole-body CT imaging, and whether the falciform fat pad weight (FFPW), adjusted to animal size, could be used to indicate degree of excess body fat.

## Materials and methods

### Sample population and experimental design

Our prospective cross-sectional study was performed postmortem and involved the use of client-owned animals presented for educational and research purposes at the Department of Biomedical Sciences and Veterinary Public Health, Swedish University of Agricultural Sciences (SLU; Uppsala, Sweden). Adult cats (≥2-y-old) euthanized within 72 h of postmortem examination were included. Whole-body CT was used as the “gold standard” to determine tBF, and we determined the body fat percentage (%BF), based on ratios of adipose tissue and lean soft tissue, and the normalized body fat volume (nBFV), based on ratios of body fat volume (BFV), and total body bone volume (TBBV). At postmortem examination, the FFPW was recorded and normalized for animal size using ratios of the FFPW and femur length (FL). In addition, subjective visual assessment of body fat was performed, and each cat was assigned to a fat category (FC) to evaluate agreement between objectively and subjectively determined postmortem fat classification. We excluded cats with primary disease of adipose tissue, generalized peritonitis, or known surgery in anatomic regions in close proximity to the falciform ligament. In addition, exclusion criteria for CT evaluation of tBF were focally extensive or generalized primary disease of skeletal musculature. Owner consent for the animal to be used in research was obtained for all cats.

### Computed tomography examination

After death, cats were kept in a refrigerated room if CT examination and postmortem examination were not performed immediately after euthanasia. Cats were positioned on a conforming foam cushion in ventral recumbency, with the front legs extended cranially, the hind legs extended caudally, and the head towards the gantry. The CT images were obtained using a third-generation, 64-slice multidetector CT scanner (Definition; Siemens). Transverse images were acquired using a helical protocol with exposure values of 250 kV, 160 mAs, focal spot 1.2 mm, spiral pitch factor 0.8, and the reconstruction diameter was adjusted individually according to the cat size. Images for fat evaluation had a soft tissue convolution kernel (B30s), slice thickness 0.6 mm, and slice increment 0.6 mm. Images for FL measurements had a high-resolution convolution kernel (B70s), slice thickness 0.6 mm, and slice increment 0.3 mm.

### Postmortem evaluation and calculation of the FFPW:FL ratio

**Figures 1, 2.**
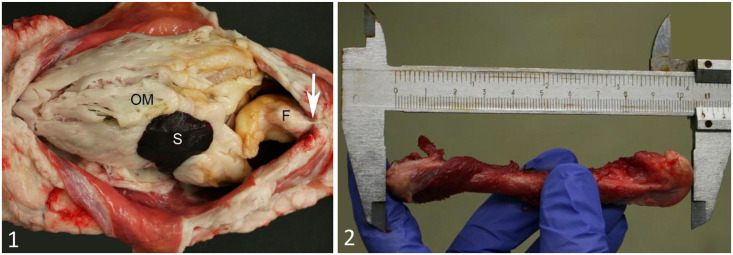
The opened abdominal cavity of a cat, and measuring a cat femur. **Figure 1.** Macroscopic appearance of the ventral aspect of the abdominal cavity of a cat. After incision of the linea alba, the falciform fat pad (F) has been separated from the abdominal wall by blunt dissection. To exteriorize the falciform fat pad, its attachment to the ventral abdominal wall at the xiphoid process (arrow) is transected. OM = greater omentum; S = spleen. **Figure 2.** Measurement of cat femur length using calipers. The greatest distance between the proximal aspect of the greater trochanter and the distal articular surface of the lateral femoral condyle is measured.

Body weight was recorded using digital scales (Ek-12KA, A&D; or FCB 12K1, Kern & Sohn; or TCS-D 200A, Vetek). After weighing, the skin was removed from the head and the body to the level of the metacarpi and metatarsi and the base of the tail. The abdominal cavity was opened through incising the linea alba. The falciform fat pad was exteriorized after separating it from the abdominal wall and the xiphoid process of the sternum (Fig. 1). The FFPW was recorded using digital scales (Ek-12KA, A&D; or FCB 12K1, Kern & Sohn). The animal was then subjected to postmortem examination, and, based on subjective visual assessments of amounts of subcutaneous, intraabdominal, retroperitoneal, and peri- and epicardial fat, each cat was assigned to a FC. FCs comprised severe underweight (complete absence of fat and presence of serous atrophy), underweight, normal weight, overweight, and obesity. The skeletal musculature was graded as atrophic, normal, or hypertrophic. Whereas the musculature was graded by one evaluator, visual fat assessments were independently performed by 1–3 evaluators.

The FL of one randomly selected femur in each cat was recorded using analog calipers (measuring range 150 mm, beak length 40 mm, Vernier scale 0.05 mm). The FL was determined by measuring the distance between the proximal margin of the major trochanter and the distal articular surface of the lateral femoral condyle (Fig. 2). For each cat, the FFR was calculated by dividing the FFPW (in g) by FL (in cm to 2 decimals).

### Calculations of tBF and bone parameters in CT images

CT images were transferred to a workstation with image viewing software (Horos, https://horosproject.org/). Window width 400 Hounsfield units (HU) and window level 40 HU were used for segmentation of soft tissue attenuation structures, and window width 4,000 HU and window level 700 HU were used for segmentation of bone and metal attenuation structures. Manual segmentation of the images was done using the “freehand pencil tool” and the “generate missing regions of interest tool.” The CT table and any objects external to the cat’s body with values >−251 HU were segmented, and values in those regions set to −1,024 HU. To prepare the images for fat, lean soft tissue, and bone measurements, the non-aerated lungs, the urine in the urinary bladder, and the contents of the large intestine and stomach were segmented and values in those regions set to −1,024 HU. In addition, to prepare images for TBBV measurements, any bone and metal attenuation structures in the cat’s body that were not part of the skeleton (e.g., microchips, surgical implants, areas of soft tissue mineralization, and urinary calculi) were segmented and values in those regions set to −1,024 HU.

Calculations were done using ImageJ software (1.44o, 64-bit, https://imagej.nih.gov/ij/index.html) and Excel (v.16.46; Microsoft). The %BF was determined using a method described previously.^5^ Briefly, frequency histogram data lists of HU values were created and voxels with values ≥−250 HU and ≤250 HU selected. In each cat, peaks for fat and lean soft tissues were identified and the HU value located at the midpoint between the 2 tissue peaks determined. Values ≥−250 HU and ≤ the mid-point HU value were considered fat attenuation voxels. Values > the midpoint HU value and ≤250 HU were considered lean soft tissue attenuation voxels.

Calculation of the nBFV required calculation of the voxel volume (VV), the BFV, and the TBBV for each cat. Frequency histogram data lists of the HU values were created from segmented bone images, and voxels with values ≥350 HU were considered bone attenuation voxels.

The %BF was calculated by the equation:

$$\%\mathrm{BF}=\frac{\mathrm{no}.\;\mathrm{of}\;\;\mathrm{fat}\;\mathrm{voxels}}{\mathrm{no}.\;\mathrm{of}\;\mathrm{fat}\;\mathrm{voxels}+\mathrm{no}.\;\mathrm{of}\;\mathrm{lean}\;\mathrm{soft}\;\mathrm{tissue}\;\mathrm{voxels}}\times 100\%$$

The VV was calculated by the equation:

$$\begin{array}{l} \mathrm{VV}\left({\mathrm{cm}}^{3}\right)=\mathrm{pixel}\;\mathrm{width}\left(\mathrm{cm}\right)\times \mathrm{pixel}\;\mathrm{length}\left(\mathrm{cm}\right)\times \\ \;\;\;\;\;\;\mathrm{slice}\;\mathrm{thickness}\left(\mathrm{cm}\right). \end{array}$$

The BFV was calculated by the equation:

$$\mathrm{BFV}\left({\mathrm{cm}}^{3}\right)=\mathrm{no}.\;\mathrm{of}\;\mathrm{fat}\;\mathrm{voxels}\times \mathrm{VV}.$$

The TBBV was calculated by the equation:

$$\mathrm{TBBV}\left({\mathrm{cm}}^{3}\right)=\mathrm{no}.\;\mathrm{of}\;\mathrm{bone}\;\mathrm{voxels}\times \mathrm{VV}.$$

The nBFV was calculated by the equation:

$$\mathrm{nBFV}=\frac{\mathrm{BFV}\left({\mathrm{cm}}^{3}\right)}{\mathrm{TBBV}\left({\mathrm{cm}}^{3}\right)}.$$

To calculate FL, CT images were aligned using 3-dimensional multiplanar reconstruction into standardized planes. The maximum distance between the proximal margin of the major trochanter and the distal articular surface of the lateral femoral condyle was measured in the sagittal plane image (Suppl. Figs. 1–5).

### Calculations of lower cutoff values for overweight and obesity, and test for FC agreement between the FFR and subjective assessment

Determination of FC for each cat was according to the visual assessment made by the evaluator performing most assessments (C. Ley, *n* = 53 assessments). The lower nBFV cutoff value for overweight was calculated as the sum of the median nBFV value for normal weight cats and the median nBFV value for overweight cats divided by 2. The lower nBFV cutoff value for obesity was calculated as the sum of the median nBFV value for overweight cats and the median nBFV value for obese cats divided by 2. The nBFV cutoff values were then used in the linear regression equations to derive the lower FFR cutoff values for overweight and obesity. After cutoff values for the FFR were determined, all cats were assigned to a FC according to the calculated FFR and results compared to the FC assignment according to visual assessment.

### Statistical analyses

Descriptive statistics were performed regarding age, sex, body weight, FFPW, FL, FFR, %BF, BFV, TBBV, and nBFV. Linear regression was used to determine correlations between body weight and BFV, between FFPW and BFV, between FL determined at postmortem examination and in CT images, between CT-determined FL and TBBV, and between FFR and nBFV. If normality and/or constant variance tests failed in analyses, transformation using the natural logarithm was used. In cases in which FL was unrecorded at postmortem examination, FL recorded in CT images was used. The means of left and right FL were used in calculations of correlations between FL and TBBV. Mean, median, minimum, maximum, and the first and the third quartiles were calculated for the nBFV and FFR for cats within the FCs underweight, normal weight, overweight, and obese. Differences in nBFV and FFR between FCs were evaluated using ANOVA on ranks; the Dunn method was used for pairwise multiple comparisons. Statistical analyses were performed using SigmaPlot 13.0 (Systat Software), and *p* ≤ 0.05 was considered significant. The sensitivity and specificity for detection of overweight and obesity using the FFR (including exact binominal 95% CIs) were calculated using online statistical software (https://statpages.info/ctab2x2.html). For obesity, FC underweight, normal weight, and overweight equaled a negative disease outcome and FC obesity a positive disease outcome, and FFR <3.5 a negative test and FFR ≥3.5 a positive test. For overweight, FC underweight, normal weight, and obesity equaled a negative disease outcome and FC overweight a positive disease outcome, and FFR <1.6 or ≥3.5 a negative test and FFR ≥1.6 or <3.5 a positive test. Sensitivity and specificity values <0.5 were classified as low, 0.5–0.69 as moderate, 0.7–0.89 as high, and 0.9–1 as very high.

## Results

### Demographic data

We included 54 cats in our study. Cats with tBF determined both with CT and at postmortem examination (*n* = 39) had a mean age of 10.7 y (range: 6–19 y). There were 26 Domestic Shorthair cats (67%), 3 Domestic Longhair cats (8%), 9 purebred cats (23%; 3 Bengal cats, 3 Norwegian Forest cats, 2 Ragdolls, 1 British Longhair), and 1 crossbred cat (3%). There were 22 castrated males (56%), 16 spayed females (41%), and 1 intact male (3%). Cats that did not have tBF determined with CT (*n* = 15) had a mean age of 10.4 y (range: 5–17 y). These cats comprised 12 Domestic Shorthair cats (80%) and 3 purebred cats (20%; 1 Bengal cat, 1 Persian cat, 1 Ocicat). There were 12 castrated males (80%), 1 spayed female (7%), 1 intact male (7%), and 1 intact female (7%; Suppl. Table 1).

### Objectively determined parameters

All 54 cats had body weights and FFPW recorded at postmortem examination (Table 1; Suppl. Table 1). In 2 cats, the FFPW was below recordable scale weight (reading on scales 0 g).

**Table 1.** Mean (minimum; maximum) values for fat and bone parameters determined by computed tomography (CT) and at postmortem examination.

	Unit	Cats with body fat evaluated with CT and at postmortem examination (*n* = 39)	Cats with body fat evaluated at postmortem examination only (*n* = 15)	All cats (*n* = 54)
BW	g	4,810 (2,850; 8,590)	4,000 (2,170; 7,700)	4,580 (2,170; 8,590)
FFPW	g	34 (3; 167)	29 (0; 170)	33 (0; 170)
FL*	cm	11.43 (10.04; 13.00)	11.01 (9.92; 12.05)	11.32 (9.92; 13.00)
%BF		33.8 (13.1; 66.6)	NA	NA
BFV	cm^3^	1,410 (375; 3,790)	NA	NA
TBBV	cm^3^	236 (153; 333)	NA	NA
nBFV		6.1 (1.8; 16.7)	NA	NA
FFR		3.0 (0.2; 14.7)	2.5 (0.0; 14.1)	2.9 (0.0; 14.7)

%BF = body fat percentage; BFV = body fat volume; BW = body weight; FFPW = falciform fat pad weight; FFR = FFPW:FL ratio; FL = femur length; NA = not applicable; nBFV = normalized BFV; TBBV = total body bone volume.

* Based on postmortem examination values, complemented by CT-determined FL if postmortem value was unrecorded.

Five cats were excluded from CT tBF determination because of the lack of defined fat peaks. These cats were all classified as underweight at postmortem examination (although one cat had an evaluator disagreement of underweight or normal weight). Hence, 39 cats were included in the analyses of CT-estimated tBF (Table 1; Suppl. Table 1). Voxel sizes in the CT images were 0.07–0.15 mm^3^ (mean 0.1 mm^3^, SD 0.02 mm^3^).

**Figures 3, 4.**
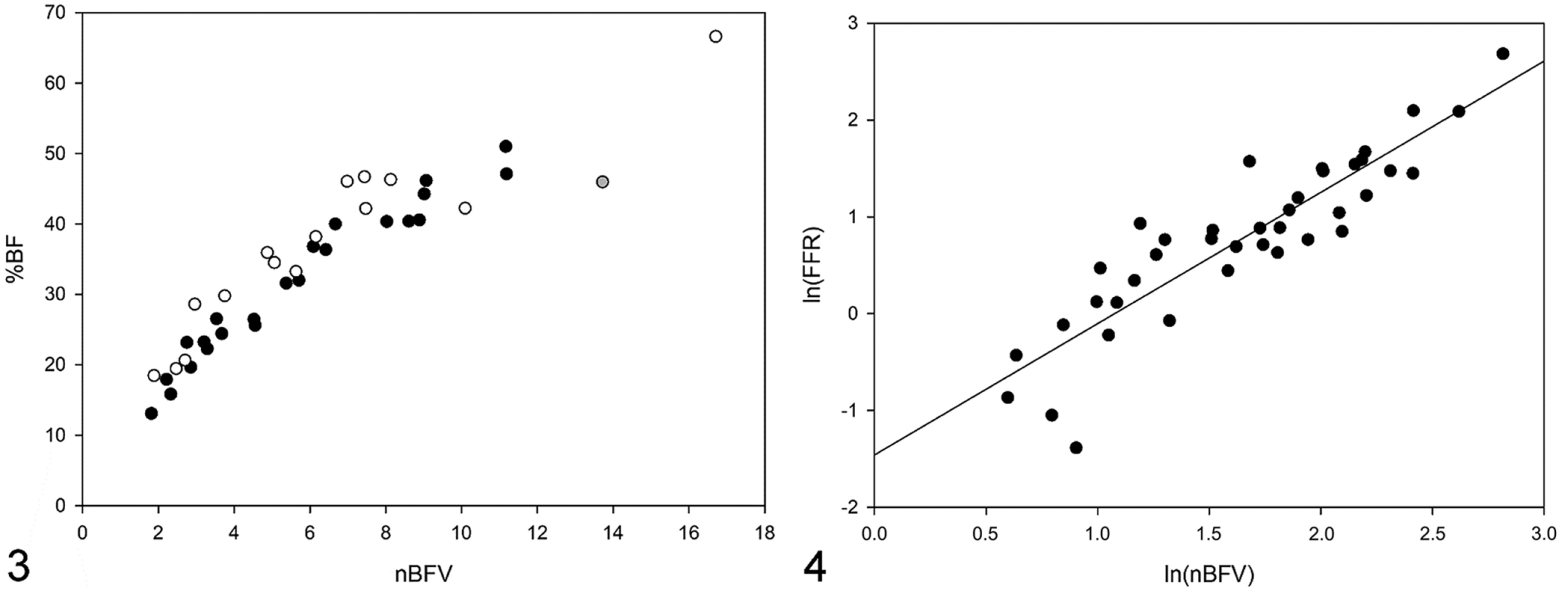
Correlations of body fat percentage and falciform fat pad weight:femur length to normalized body fat volume. **Figure 3.** There was very strong correlation between the body fat percentage (%BF) and normalized body fat volume (nBFV; *n* = 39 cats; *p* < 0.001, *r* = 0.931, *r*^2^ = 0.867, regression equation %BF = 14.396 + [3.199 × nBFV]). When cats with muscle atrophy (*n* = 15, open circles) and hypertrophy (*n* = 1, gray-filled circle) were excluded, the correlation was even stronger (*p* < 0.001, *r* = 0.974, *r*^2^ = 0.949, regression equation %BF = 10.697 + [3.655 × nBFV]). **Figure 4.** There was very strong correlation between the falciform fat pad weight:femur length ratio (FFR) and nBFV (*p* < 0.001, *r* = 0.897, *r*^2^ = 0.805, regression equation ln(FFR) = −1.459 + [1.356 × ln(nBFV)]). Values were transformed to the natural logarithm given the failed normality test. Solid line in graph is the regression line.

There was strong correlation between body weight and BFV (*p* < 0.001, *r* = 0.782, *r*^2^ = 0.611) and between FFPW and BFV (*p* < 0.001, *r* = 0.888, *r*^2^ = 0.788). There was very strong correlation between %BF and nBFV (*p* < 0.001, *r* = 0.931, *r*^2^ = 0.867), and the correlation was even stronger when cats with muscle atrophy or hypertrophy were excluded from the analysis (*p* < 0.001, *r* = 0.974, *r*^2^ = 0.949, remaining cats *n* = 23; Fig. 3).

The FL was recorded at postmortem examination in 43 of 54 cats (80%) and in CT images in 45 of 54 cats (83%; Table 1, Suppl. Table 1). In one cat, only a CT image of the left femur was available for FL measurement. In 34 of 43 femurs (79%) measured at postmortem examination, FL measurements were also performed in CT images. There was near-perfect correlation between FL recorded at postmortem examination and in CT images (*p* < 0.001, *r* 0.999, *r*^2^ = 0.997) and between the left and right FL in CT images (*p* < 0.001, *r* = 0.998, *r*^2^ = 0.996). There was strong correlation between CT-determined FL and TBBV (*p* < 0.001, *r* = 0.879, *r*^2^ = 0.772) and strong correlation between FFR and nBFV (*p* < 0.001, *r* = 0.897, *r*^2^ = 0.805; Fig. 4).

**Figures 5–7.**
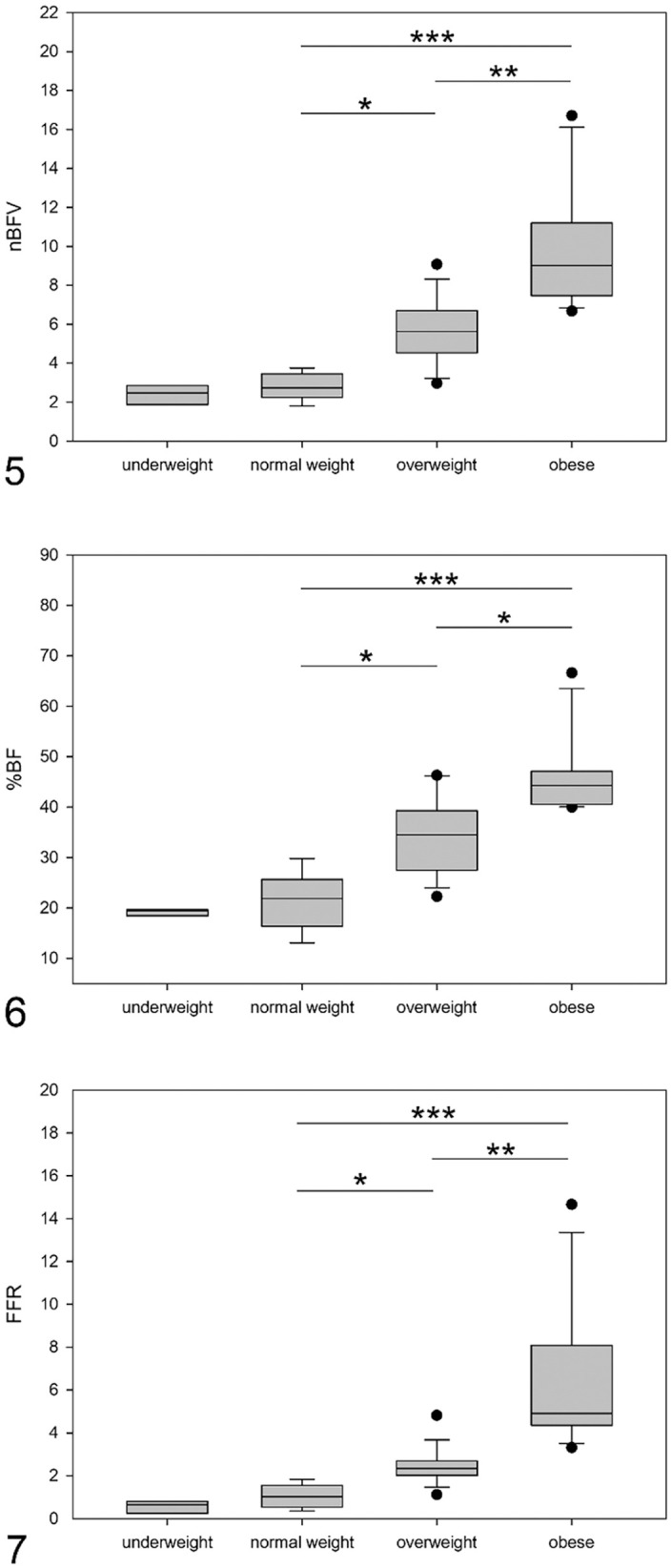
Boxplot graphs of normalized body fat volume (nBFV), body fat percentage (%BF), and falciform fat pad weight:femur length ratio (FFR) to fat categories in underweight (*n* = 3), normal weight (*n* = 8), overweight (*n* = 17), and obese (*n* = 11) cats. **Figure 5.** Boxplot graph of nBFV in the 4 fat categories. The nBFV was higher in obese compared to overweight and normal weight cats (*p* = 0.007 [**] and *p* < 0.001 [***], respectively) and in overweight compared to normal weight cats (*p* = 0.021 [*]). **Figure 6.** Boxplot graph of %BF in the 4 fat categories. The %BF was higher in obese compared to overweight and normal weight cats (*p* = 0.016 [*] and *p* < 0.001 [***], respectively) and in overweight compared to normal weight cats (*p* = 0.027 [*]). **Figure 7.** Boxplot graph of FFR in the 4 fat categories. The FFR was higher in obese compared to overweight and normal weight cats (*p* = 0.003 [**] and *p* < 0.001 [***], respectively) and in overweight compared to normal weight cats (*p* = 0.028 [*]).

The nBFV was higher in obese cats compared to overweight and normal weight cats (*p* = 0.007 and *p* < 0.001, respectively), and in overweight cats compared to normal weight cats (*p* = 0.021; Table 2, Fig. 5). Similarly, the %BF was higher in obese compared to overweight and normal weight cats (*p* = 0.016 and *p* < 0.001, respectively) and in overweight compared to normal weight cats (*p* = 0.027; Table 2, Fig. 6). The FFR was higher in obese cats compared to overweight and normal weight cats (*p* = 0.003 and *p* < 0.001, respectively) and in overweight compared to normal weight cats (*p* = 0.028; Table 2, Fig. 7). Absence of definable CT fat peaks in several underweight cats resulted in this group comprising only 3 cats. Given the low number of cats, this category was excluded from the ANOVA on rank analyses.

**Table 2.** Mean, SD, 1st and 3rd quartiles (Q1, Q3) for the normalized body fat volume (nBFV), body fat percentage (%BF), and falciform fat pad weight:femur length ratio (FFR) in cats with body fat evaluated by computed tomography and postmortem examination (*n* = 39).

	Underweight (*n* = 3 cats)	Normal weight (*n* = 8 cats)	Overweight (*n* = 17 cats)	Obese (*n* = 11 cats)
nBFV				
Mean ± SD	2.4 ± 0.5	2.8 ± 0.7	5.7 ± 1.7	10.1 ± 3.0
Median (Q1, Q3)	2.5 (1.9, 2.8)	2.7 (2.2, 3.4)	5.6 (4.5, 6.7)	9.0 (7.5, 11.2)
%BF				
Mean ± SD	19.2 ± 0.6	21.3 ± 5.5	34.4 ± 7.6	46.1 ± 7.6
Median (Q1, Q3)	19.5 (18.5, 19.6)	21.9 (13.1, 29.8)	34.5 (22.3, 46.3)	44.3 (40.0, 66.6)
FFR				
Mean ± SD	0.6 ± 0.3	1.1 ± 0.5	2.4 ± 0.8	6.2 ± 3.2
Median (Q1, Q3)	0.6 (0.2, 0.8)	1.0 (0.5, 1.6)	2.3 (2.0, 2.7)	4.9 (4.4, 8.1)

### Cutoff values for overweight and obesity

The lower cutoff value for nBFV for overweight was 4.180 and for obesity 7.322. Based on the linear regression equation for nBFV versus %BF in cats with muscle atrophy or hypertrophy excluded (%BF = 10.697 + [3.655 × nBFV]), the lower cutoff values for obesity and overweight were calculated for the %BF. The lower cutoff value for obesity corresponded to %BF 37.4, and the lower cutoff value for overweight corresponded to %BF 27.1.

Based on the nBFV versus FFR linear regression equation, the lower cutoff values for obesity and overweight were calculated for the FFR as ln(FFR) = −1.459 + (1,356 × ln[7.322]) and ln(FFR) = −1.459 + (1.356 × ln[4.180]), respectively. By using these equations, the lower cutoff value for obesity corresponded to an FFR of 3.5, and the lower cutoff value for overweight corresponded to an FFR of 1.6. The sensitivity and specificity for obesity was 0.93 (95% CI: 0.66–1.00) and 0.95 (95% CI: 0.83–0.99), respectively. The sensitivity and specificity for overweight was 0.85 (95% CI: 0.62–0.97) and 0.91 (95% CI: 0.76–0.98), respectively.

### Subjective assessment findings at postmortem examination

Forty-two of 54 cats (78%) were subjectively assessed by 2 evaluators, 2 of 54 cats (4%) by 3 evaluators, and 10 of 54 (18%) by 1 evaluator. There was agreement between evaluators regarding the FC in 37 of 44 cats (84%) and disagreement in 7 of 44 cats (16%; Suppl. Table 1). Fourteen of 54 cats (26%) were classified as obese (including 1 cat with evaluator disagreement), 20 of 54 (37%) as overweight (including 3 cats with evaluator disagreement), 11 of 54 (20%) as normal weight (including 1 cat with evaluator disagreement), and 9 of 54 (17%) as underweight (including 2 cats with evaluator disagreement). No cat was classified as severely underweight. The skeletal musculature was graded normal in 29 of 54 cats (54%), atrophic in 24 of 54 cats (44%), and hypertrophic in 1 of 54 cats (2%; Suppl. Table 1).

### Agreement in FC classification using the FFR and subjective assessment

There was a discrepancy in FC classification in 6 of 54 (11%) cats using the FFR cutoff values 1.6 ≥ overweight <3.5, and obese ≥3.5 compared to visual subjective assessment, and in 3 of these there was evaluator disagreement for visual assessment. Two obese cats (FFR 4.8 and FFR 3.8, respectively) were classified as overweight using visual assessment (evaluator agreements). One overweight cat (FFR 3.3) was classified as obese (evaluator disagreement obese or overweight), and one overweight cat (FFR 1.8) was classified as normal weight (evaluator disagreement normal weight or overweight). One further borderline overweight cat (FFR 1.6) was classified as normal weight (one evaluator only) and one normal weight cat (FFR 1.1) was classified as overweight (evaluator disagreement normal weight or overweight). For the 3 cats with evaluator disagreement, evaluation protocol notes revealed that one evaluator considered the FC borderline between categories. No cats classified as underweight on visual assessment had an FFR of ≥1.6.

## Discussion

We explored the FFPW as a method for objective estimation of tBF in routine postmortem examination of cats, using whole-body CT as the “gold standard” for tBF estimation. We found that the FFPW was strongly correlated to the tBF as determined by the BFV. This is in accordance to results from a study using MRI, which showed equal distribution of the abdominal fat mass between subcutaneous and intra-abdominal areas.^11^ To use the FFPW as an indicator of tBF, an adjustment for cat size was made by dividing the FFPW by the FL. The strong correlations between both FFPW and BFV, and FFR and nBFV, suggest that this fat deposit is useful for objective tBF estimation at postmortem examination, and, when cats were grouped according to FC, there were significant differences among normal, overweight, and obese animals for both the nBFV and the FFR. Further, the sensitivity and specificity for detecting obesity using the FFR were very high, and, for overweight, the sensitivity was high and the specificity very high. This suggests that the proposed FFR cutoff values are useful for postmortem detection of obesity and overweight in cats.

Whole-body CT has been used to evaluate tBF in cats by determining the %BF,^3,5,19,25^ and has been found to give similar information to the traditional “gold standard” method DEXA.^5^ Using CT, we estimated tBF both as the %BF and by using the nBFV. There was very strong correlation between the %BF and nBFV, indicating agreement in tBF estimates between the 2 methods. The even stronger correlation between the %BF and nBFV seen when animals with muscle atrophy and hypertrophy were excluded is explained mainly by the impact of variations in lean soft tissue volumes in calculations of %BF. In calculations of the %BF, a large proportion of the lean soft tissue is represented by skeletal muscle. Amounts of lean soft tissue may depend on the individual’s living conditions, physical activity, and age, and muscle wasting is commonly present in animals suffering from serious disease.^2,8,23^ Twenty-four of the 54 examined cats in our study had muscle atrophy, which suggests that muscle atrophy is common in cats presented for postmortem examination. Hence, muscle state is important to take into consideration in tBF estimation using %BF, and suggests that a more robust parameter, such as bone length and bone volumes, is valuable to use rather than lean soft tissue when normalizing amounts of tBF in diseased animals.

The correlation between body weight and BFV was strong, however, less than that for the FFPW and BFV. This is not surprising given that confounding factors that influence body weight include animal size, weight of ingesta in the gastrointestinal tract, urine in the urinary bladder, and fluid in body cavities. It suggests that body weight should be avoided as an indicator of tBF, in preference to using more reliable parameters based on amounts of fat.

We used the FL as a reference for body size and found no difference in length between right and left femurs. This agrees with a study of the feline ulna and radius,^20^ and suggests that either the left or right femur can be used in FFR calculations. By using the greater trochanter as one of the measuring points, interference from articular changes, such as osteophyte formation, was avoided. If remodeling of the lateral condyle joint surface is present, FL may be difficult to determine accurately. We noted no interference on FL measurements by disease processes. The near-perfect correlation between FL recorded at postmortem examination and in CT images suggests that this measurement is reliable. However, it is worth noting that the FFR could give a misleading value in cases in which a disease process or surgery involves the falciform fat pad or the femur. In addition, the need to dissect out the femur to measure the FL means that the FFR method would not be suitable for cosmetic postmortem examinations, and this is a limitation for its use.

Based upon the median values of the nBFV in different FCs, we propose that an FFR between 1.6 and <3.5 suggests overweight, and an FFR of ≥3.5 suggests obesity. Nevertheless, it is worth considering that the cutoff values were based on median values, and these were dependent on the cohort characteristic in regard to excess body fat. It is possible that cutoff values for overweight and obesity would be different in another study population, and it would be valuable to perform further studies using larger numbers of cats to strengthen the accuracy of the proposed cutoff values. Additionally, to establish the cutoff values for the nBFV, we referred to the assignment of cats according to FC classification, which in turn was determined by visual assessment. However, we believe that there currently is no more reliable alternative to visual assessment that could have been used.

All but 2 cats in our study that had CT measurements were neutered, and there were more castrated males than spayed females. Statistical analyses to investigate differences in tBF between neutered males and females was not performed because the study population was heterogeneous in regard to age distribution, breed, and presence of disease. However, a study in healthy cats has shown that there is no difference in %BF between castrated males and spayed females within a body condition score (BCS) category.^3^ Given the strong correlation between %BF and nBFV that we found, it can be assumed that there similarly will be no significant difference in nBFV between castrated male and spayed female cats within the same BCS and that the method described here will be applicable for both castrated male and spayed female cats. Given the paucity of non-neutered cats, conclusions regarding the FFR in non-neutered animals cannot be made.

A study that evaluated the agreement between a clinical 9-point BCS system and %BF showed that the %BF for the same BCS was higher for neutered indoor cats^3^ than previously shown in a group of non-neutered colony cats.^13^ We did not compare %BF or nBFV to a BCS because we considered that this would be less reliable than using the FC in regard to reflecting amounts of tBF. However, we found that the mean %BF value for overweight was 34.4 and for obesity 46.1, and that these values are similar to those reported for neutered indoor cats in BCS 6 (34.6 for castrated male cats and 39.3 for spayed female cats) and in BCS 8 (46.9 for castrated male cats and 48.6 for spayed female cats), respectively.^3^

To our knowledge, the use of CT or DEXA for postmortem evaluation of tBF in cats has not been described. Unlike 2-dimensional DEXA, CT is a 3-dimensional imaging method, and it is possible to remove regions of the images that are not true components of the cat’s body composition, including ingesta, feces, and urine. This makes CT theoretically a more robust method than DEXA for estimating body composition, particularly postmortem, when animals are submitted for evaluation without fasting, and may have large volumes of urine in the urinary bladder and ingesta in the gastrointestinal tract. In our study, contents in the stomach, large intestine, and urinary bladder were removed using manual image segmentation. In addition, non-aerated lung regions were removed from the images using manual segmentation because atelectatic postmortem lung has attenuation values in the range of both fat and lean soft tissues. The use of manual segmentation can be seen as a limitation of our study given that it relies on subjective judgement and manual drawing of regions of interest. However, the variation in gastrointestinal tract, urinary bladder filling, and lung atelectasis meant that simple automated segmentation was not possible, and we believe that the advantages of segmentation outweigh the potential disadvantages. Further limitations of CT for determination of tBF include partial volume averaging artefacts and the division of fat attenuation voxels and lean tissue voxels. When focal collections of fat tissue are smaller than the voxel size or when fat tissue only fills part of a voxel, then partial volume averaging will occur, and this may result in this fat tissue not being included in the fat volume calculation. The mean voxel size in our study was 0.1 mm^3^, which is small and should minimize the effect of partial volume averaging; however, focal microscopic fat tissue within lean soft tissue or bone may not be detected with the CT method.

The division of fat attenuation voxels and lean tissue voxels in CT images relied on identification of fat and lean soft tissue peaks in the frequency histogram of the voxel HU values following a previously described calculation method with good correlation to DEXA.^5^ These peaks were easily identified in normal, overweight, and obese cats, but fat peaks were often not defined in underweight cats. This resulted in several underweight cats being excluded from CT fat evaluation. It would have been optimal to have been able to determine the tBF in all of the underweight cats. Other calculation methods for fat evaluation from CT images have been described in cats.^5^ However, these have a lower correlation with DEXA and, given that our study was focused on detection of excess body fat, other calculation methods for these underweight cats were not attempted.

The procedure to determine the FFR was straightforward and technically unchallenging, and the simplicity of the method makes it suitable for use in routine postmortem examinations. The falciform fat pad was easy to identify and exteriorize, and the femur was easy to remove from the body and measure. Including the FFR in feline postmortem protocols provides a way to standardize postmortem fat evaluation in cats and has the potential to reduce inter-observer variability regarding classification of overweight and obesity.

## Supplemental Material

sj-pdf-1-vdi-10.1177_10406387211071078 – Supplemental material for Falciform fat:femur length ratio provides a novel method for objective postmortem estimation of total body fat in overweight and obese cats Click here for additional data file. Supplemental material, sj-pdf-1-vdi-10.1177_10406387211071078 for Falciform fat:femur length ratio provides a novel method for objective postmortem estimation of total body fat in overweight and obese cats by Cecilia Ley, Alexandra T. Leijon, Tora E. Nyberg, Lisa M. Lindström and Charles J. Ley in Journal of Veterinary Diagnostic Investigation
