# Supplementary material for: Falciform fat:femur length ratio provides a novel method for objective postmortem estimation of total body fat in overweight and obese cats
Source: J Vet Diagn Invest. 2022 Jan 10;34(2):237–45. doi: 10.1177/10406387211071078 (PMC8915224; doi:10.1177/10406387211071078)
Supplement: sj-pdf-1-vdi-10.1177_10406387211071078 – Supplemental material for Falciform fat:femur length ratio provides a novel method for objective postmortem estimation of total body fat in overweight and obese cats [file sj-pdf-1-vdi-10.1177_10406387211071078.pdf]

Ley C, et al. Falciform fat:femur length ratio provides a novel method for objective postmortem estimation of total body fat in overweight and obese cats

Supplemental Table 1. Raw data from 54 adult cats examined by computed tomography (CT) and/or at postmortem (PM) examination.

Cat ID	Age (y)‡	Sex	Breed	BW (g)	FFPW (g)	VFA	MG	%BF	BFV	TBBV	nBFV	FL, cm (R/L, method)	FFR
**Cats with body fat evaluation on CT and at postmortem examination (*n* = 39)**													
FC OB, evaluator agreement or only one evaluator													
3	13	SF	BEN	5,380	88	OB (EV1)<br>OB (EV3)	N	47.1	2,240	200	11.2	10.80 (R, CT)<br>10.71 (L, CT)	8.2
10	11	CM	CRS	4,120	48	OB (EV1)<br>OB (EV2)	A	46.7	1,590	214	7.4	10.72 (R, PM)<br>10.76 (R, CT)<br>10.77 (L, CT)	4.5
23	7	SF	NorFC	6,820	50	OB (EV1)	A	42.2	2,460	244	10.1	11.43 (R, PM)<br>11.58 (R, CT)<br>11.51 (L, CT)	4.4
24	11	SF	DSH	4,250	48	OB (EV1)<br>OB (EV2)	N	40.4	1,460	169	8.6	10.25 (R, PM)<br>10.28 (R, CT)<br>10.33 (L, CT)	4.7
28	9	SF	DLH	5,080	51	OB (EV1)<br>OB (EV4)	N	40.6	1,780	200	8.9	10.38 (R, PM)<br>10.28 (R, CT)<br>10.35 (L, CT)	4.9

Cat ID	Age (y)‡	Sex	Breed	BW (g)	FFPW (g)	VFA	MG	%BF	BFV	TBBV	nBFV	FL, cm (R/L, method)	FFR
41	10	CM	DSH	5,100	47	OB (EV1)<br>OB (EV4)	N	51.0	2,240	200	11.2	11.02 (R, PM)<br>11.08 (R, CT)<br>11.10 (L, CT)	4.3
44	13	CM	DSH	5,080	51	OB (EV1)<br>OB (EV4)	A	42.2	1,820	243	7.5	11.69 (R, PM)<br>11.62 (R, CT)<br>11.53 (L, CT)	4.4
45	13	CM	DSH	6,060	61	OB (EV1)<br>OB (EV4)	N	44.3	2,330	259	9.0	11.44 (L, PM)<br>11.40 (R, CT)<br>11.41 (L, CT)	5.3
46	9	CM	DSH	6,280	167	OB (EV1)<br>OB (EV4)	A	66.6	3,790	227	16.7	11.39 (L, PM)<br>11.44 (R, CT)<br>11.34 (L, CT)	14.7
54	6	M	DSH	8,590	89	OB (EV1)<br>OB (EV4)	H	46.0	3,480	254	13.7	11.02 (R, PM)<br>11.04 (R, CT)<br>11.02 (L, CT)	8.1
FC OW, evaluator agreement or only one evaluator													
2	13	CM	NorFC	4,160	23	OW (EV1)<br>OW (EV2)	A	34.5	1,170	232	5.1	11.50 (R, CT)<br>11.49 (L, CT)	2.0
4	9	SF	DSH	4,700	26	OW (EV1)<br>OW (EV2)<br>OW (EV3)	N	25.6	1,010	222	4.6	10.98 (R, CT)<br>10.93 (L, CT)	2.4
5	8	CM	Ben	5,180	55	OW (EV1)<br>OW (EV3)	N	31.6	1,350	252	5.4	11.40 (R, CT)<br>11.41 (L, CT)	4.8
7	18	CM	DSH	3,550	17	OW (EV1)<br>OW (EV3)	A	36.0	1,030	211	4.9	10.90 (R, CT)<br>10.89 (L, CT)	1.6

Cat ID	Age (y)‡	Sex	Breed	BW (g)	FFPW (g)	VFA	MG	%BF	BFV	TBBV	nBFV	FL, cm (R/L, method)	FFR
9	7	CM	DSH	5,910	30	OW (EV1)<br>OW (EV2)	A	33.3	1,630	290	5.6	12.42 (R, PM)<br>12.46 (R, CT)<br>12.48 (L, CT)	2.4
13	6	SF	DLH	4,010	21	OW (EV1)<br>OW (EV2)	N	36.8	1,190	195	6.1	11.18 (R, PM)<br>11.17 (R, CT)<br>11.12 (L, CT)	1.9
17	7	CM	DSH	5,280	26	OW (EV1)<br>OW (EV2)	N	24.4	1,020	277	3.7	12.09 (R, PM)<br>12.10 (R, CT)<br>12.06 (L, CT)	2.2
20	8	CM	DSH	5,890	32	OW (EV1)<br>OW (EV2)	N	22.3	1,060	321	3.3	12.59 (R, PM)<br>12.56 (R, CT)<br>12.52 (L, CT)	2.5
26	7	SF	Rag	4,840	34	OW (EV1)<br>OW (EV2)	N	40.4	1,640	204	8.0	11.96 (R, PM)<br>12.00 (R, CT)<br>11.87 (L, CT)	2.8
27	10	SF	DSH	3,610	21	OW (EV1)	N	32.0	960	168	5.7	10.31 (R, PM)<br>10.34 (R, CT)<br>10.39 (L, CT)	2.0
29	8	CM	Rag	4,860	28	OW (EV1)<br>OW (EV4)	A	46.0	1,870	268	7.0	13.00 (L, PM)<br>13.03 (R, CT)<br>12.97 (L, CT)	2.2
31	14	SF	DSH	3,500	34	OW (EV1)<br>OW (EV4)	N	46.2	1,390	153	9.1	10.04 (L, PM)<br>10.13 (R, CT)<br>10.03 (L, CT)	3.4

Cat ID	Age (y)‡	Sex	Breed	BW (g)	FFPW (g)	VFA	MG	%BF	BFV	TBBV	nBFV	FL, cm (R/L, method)	FFR
35	14	CM	DLH	6,580	31	OW (EV1)<br>OW (EV4)	A	38.2	2,050	333	6.2	12.74 (R, PM)<br>12.76 (R, CT)<br>12.77 (L, CT)	2.4
37	9	CM	NorFC	6,470	29	OW (EV1)<br>OW (EV4)	A	46.3	2,550	313	8.1	12.38 (L, PM)<br>12.38 (R, CT)<br>12.35 (L, CT)	2.3
42	7	SF	DSH	4,310	24	OW (EV1)<br>OW (EV4)	N	26.4	923	204	4.5	11.07 (R, PM)<br>11.07 (R, CT)<br>10.98 (L, CT)	2.2
FC NW, evaluator agreement or only one evaluator													
6	7	CM	DSH	4,700	5	NW (EV1)<br>NW (EV3)	N	13.1	471	259	1.8	11.93 (R, CT)<br>11.92 (L, CT)	0.4
21	6	SF	DSH	5,100	10	NW EV1)	N	15.8	640	275	2.3	11.26 (R, PM)<br>11.25 (R, CT)<br>11.22 (L, CT)	0.9
38	6	CM	DSH	4,930	16	NW (EV1)<br>NW (EV4)	N	23.2	794	248	3.2	11.33 (L, PM)<br>11.48 (R, CT)<br>11.41 (L, CT)	1.4
40	10	SF	BLH	3,940	4	NW (EV1)<br>NW (EV4)	N	17.9	538	243	2.2	11.30 (R, PM)<br>11.32 (R, CT)<br>11.30 (L, CT)	0.4
43	19	CM	DSH	4,430	14	NW (EV1)<br>NW (EV4)	A	20.6	714	264	2.7	12.37 (L, PM)<br>12.38 (R, CT)<br>12.37 (L, CT)	1.1

Cat ID	Age (y)‡	Sex	Breed	BW (g)	FFPW (g)	VFA	MG	%BF	BFV	TBBV	nBFV	FL, cm (R/L, method)	FFR
47	18	SF	DSH	3,080	10	NW (EV1)	A	29.8	738	197	3.8	10.75 (R, CT)<br>10.70 (L, CT)	0.9
53	13	CM	Ben	3,730	19	NW (EV1)	N	23.2	678	246	2.8	11.90 (R, PM)<br>11.90 (R, CT)<br>11.96 (L, CT)	1.6
FC UW, evaluator agreement or only one evaluator													
34	16	SF	DSH	2,850	7	UW (EV1)	A	18.5	375	199	1.9	10.77 (L, PM)<br>10.81 (R, CT)<br>10.75 (L, CT)	0.6
48	13	CM	DLH	4,020	3	UW (EV1)<br>UW (EV4)	A	19.5	602	244	2.5	12.04 (R, PM)<br>12.14 (R, CT)<br>12.02 (L, CT)	0.2
FC, evaluator disagreement													
32	6	SF	DSH	3,900	9	UW (EV1)<br>NW (EV4)	N	19.6	619	217	2.8	11.30 (R, PM)<br>11.33 (R, CT)<br>11.32 (L, CT)	0.8
39	12	CM	DSH	4,890	39	OB (EV1)<br>OW (EV4)	N	40.0	1,640	246	6.7	11.79 (R, CT)<br>11.76 (L, CT)	3.3
50	18	CM	DSH	3,640	14	OW (EV1)<br>NW (EV4)	A	28.6	828	279	3.0	12.45 (L, PM)<br>12.49 (R, CT)<br>12.44 (L, CT)	1.1
51	14	SF	DSH	3,020	19	NW (EV1)<br>OW (EV4)	N	26.5	633	179	3.5	10.30 (R, PM)<br>10.33 (R, CT)<br>10.29 (L, CT)	1.8

Cat ID	Age (y)‡	Sex	Breed	BW (g)	FFPW (g)	VFA	MG	%BF	BFV	TBBV	nBFV	FL, cm (R/L, method)	FFR
52	12	CM	DSH	5,660	34	OW (EV1)<br>OB (EV4)	N	36.4	1,740	271	6.4	11.64 (R, CT)<br>11.63 (L, CT)	2.9
**Cats with body fat evaluation at postmortem examination only (*n* = 15)**													
FC OB, evaluator agreement or only one evaluator													
Cat ID	Age (y)‡	Sex	Breed	BW (g)	FFPW (g)	VFA	MG	%BF	BFV	TBBV	nBFV	FL, cm (R/L, method)	FFR
1*	5	CM	DSH	6,330	81	OB (EV1)<br>OB (EV2)<br>OB (EV3)	N	NE	NE	NE	NE	11.56 (L, CT)	7.0
16	7	CM	DSH	7,700	170	OB (EV2)	N	NE	NE	NE	NE	12.05 (R, PM)	14.1
18	6	CM	DSH	3,650	75	OB (EV1)<br>OB (EV2)	A	NE	NE	NE	NE	11.12 (R, PM)	5.1
FC OW, evaluator agreement or only one evaluator													
11	11	CM	Per	4,100	39	OW (EV1)<br>OW (EV2)	A	NE	NE	NE	NE	10.21 (R, PM)	3.8
15	14	CM	DSH	3,550	19	OW (EV1)<br>OW (EV4)	A	NE	NE	NE	NE	11.16 (R, PM)	1.7
FC NW, evaluator agreement or only one evaluator													
8	6	CM	DSH	3,340	6	NW (EV1)<br>NW (EV2)	N	NE	NE	NE	NE	10.90 (X, PM)	0.6
14	11	CM	DSH	3,740	5	NW (EV1)<br>NW (EV2)	N	NE	NE	NE	NE	11.12 (R, PM)	0.4
25	6	CM	DSH	4,740	9	NW (EV1)<br>NW (EV2)	N	NE	NE	NE	NE	11.92 (R, PM)	0.8

Cat ID	Age (y)‡	Sex	Breed	BW (g)	FFPW (g)	VFA	MG	%BF	BFV	TBBV	nBFV	FL, cm (R/L, method)	FFR
FC UW, evaluator agreement or only one evaluator													
19	14	CM	Oci	2,620	1	UW (EV1)<br>UW (EV2)	A	NE	NE	NE	NE	11.16 (R, PM)	0.1
22†	13	CM	DSH	2,170	2	UW (EV1)	A	NE	NE	NE	NE	10.14 (R, PM)<br>10.18 (R, CT)<br>10.24 (L, CT)	0.2
30†	15	SF	DSH	2,670	2	UW (EV1)	A	NE	NE	NE	NE	10.74 (R, PM)<br>10.78 (R, CT)<br>10.75 (L, CT)	0.2
33†	9	CM	Ben	3,490	4	UW (EV1)	A	NE	NE	NE	NE	11.54 (L, PM)<br>11.61 (R, CT)<br>11.54 (L, CT)	0.4
36†	17	F	DSH	2,890	0	UW (EV1)<br>UW (EV4)	A	NE	NE	NE	NE	9.92 (R, CT)<br>9.84 (L, CT)	0.0
FC, evaluator disagreement													
12	9	CM	DSH	4,620	33	OW (EV1)<br>OB (EV2)	N	NE	NE	NE	NE	10.21 (R, PM)	3.2
49†	13	M	DSH	4,380	0	UW (EV1)<br>NW (EV4)	A	NE	NE	NE	NE	11.40 (L, PM)<br>11.38 (R, CT)<br>11.40 (L, CT)	0.0

A = atrophy; Ben = Bengal cat; %BF = body fat percentage; BFV = body fat volume; BLH = British Longhair; BW = body weight; CM = castrated male; CRS = crossbred; DLH = Domestic Longhair cat; DSH = Domestic Shorthair cat; EV1–4 = evaluator 1 to 4; F = female; FC = fat category at postmortem examination; FFPW = falciform fat pad weight; FFR = FFPW:FL ratio; FL = femur length; H = hypertrophy; L = left; M

= male; MG = muscle grade; N = normal; nBFV = normalized body fat volume; NE = not evaluated; NorFC = Norwegian Forest cat; NW = normal weight; OB = obese; Oci = Ocicat; OW = overweight; Per = Persian; R = right; Rag = Ragdoll; SF = spayed female; TBBV = total body bone volume; UW = underweight; VFA = visual fat assessment; X = missing data.

* CT of left femur only.

† Body fat not evaluated on CT given lack of definable fat peak.

‡ All cat ages rounded to nearest whole year (calculations made using https://www.timeanddate.com).

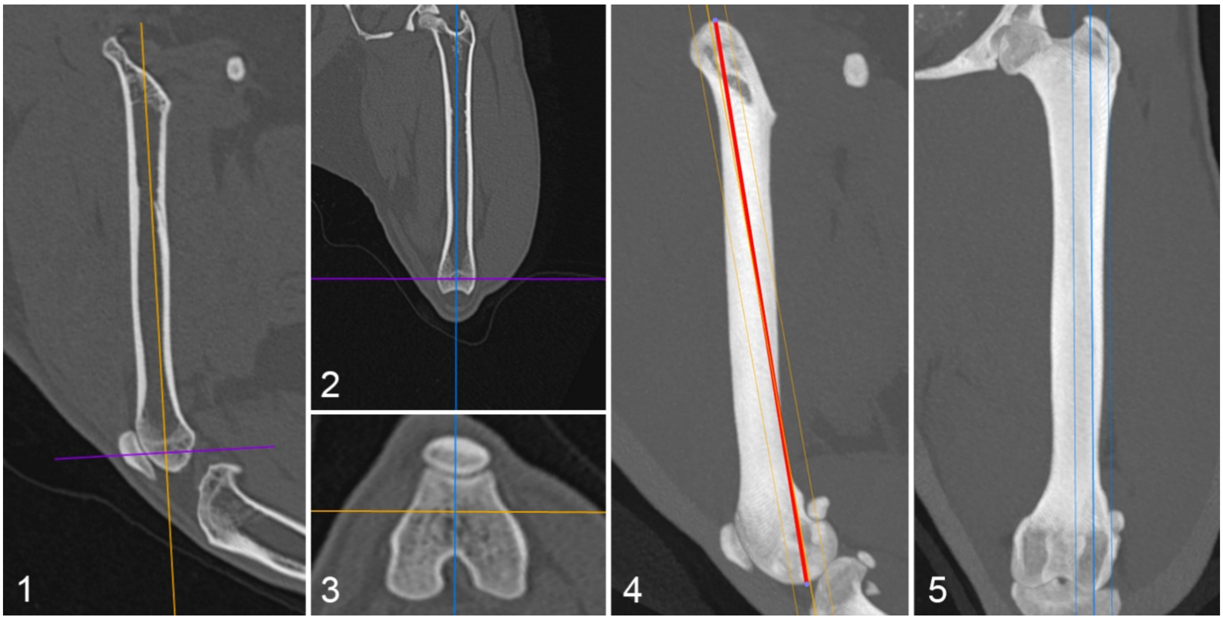

Supplemental Figures 1–5. Multiplanar reconstruction computed tomography (CT) images of the right femur of a cat showing standardized positioning of sagittal (**Figure 1**), frontal (**Figure 2**) and transverse (**Figure 3**) image planes, and the measurement of femur length (**Figures 4, 5**). Sagittal (**Figure 4**) and frontal (**Figure 5**) image planes in which the thickness of the images has been increased using a maximum intensity projection such that the sagittal plane image included the entire major trochanter and the entire lateral condyle. The sagittal and frontal image planes were then moved and angled so that the sagittal image passed from the proximal margin of the major trochanter to the distal aspect of the lateral condyle of the femur. The maximum distance (red line, Fig. 4) from the proximal margin of the major trochanter to the distal articular surface of the lateral condyle was measured in the sagittal plane image. The yellow lines (Figs. 1, 3, 4) represent the frontal plane, the purple lines (Figs. 1, 2) represent the transverse plane, and the blue lines (Figs. 2, 3, 5) represent the sagittal plane.
